# Editorial Perspective: Maximising the benefits of intervention research for children and young people with developmental language disorder (DLD) – a call for international consensus on standards of reporting in intervention studies for children with and at risk for DLD

**DOI:** 10.1111/jcpp.13694

**Published:** 2022-09-20

**Authors:** Pauline Frizelle, Cristina McKean, Patricia Eadie, Susan Ebbels, Silke Fricke, Laura M. Justice, Sari Kunnari, Suze Leitão, Angela T. Morgan, Natalie Munro, Carol‐Anne Murphy, Holly L. Storkel, Amanda Owen Van Horne

**Affiliations:** ^1^ Department of Speech and Hearing Sciences University College Cork Cork Ireland; ^2^ Department of Speech and Language Sciences, School of Education, Communication & Language Sciences Newcastle University Newcastle upon Tyne UK; ^3^ Melbourne Graduate School of Education University of Melbourne Melbourne Vic. Australia; ^4^ Moor House Research and Training Institute, Moor House School & College London UK; ^5^ Division of Psychology and Language Sciences University College London London UK; ^6^ Division of Human Communication Sciences, Health Sciences School The University of Sheffield Sheffield UK; ^7^ Department of Educational Studies The Ohio State University Columbus OH USA; ^8^ Research Unit of Logopedics, Faculty of Humanities University of Oulu Oulu Finland; ^9^ Curtin School of Allied Health Curtin University Perth WA Australia; ^10^ Speech and Language Murdoch Children's Research Institute Melbourne Vic. Australia; ^11^ Department of Audiology and Speech Pathology University of Melbourne Melbourne Vic. Australia; ^12^ Speech Pathology Department Royal Children's Hospital Melbourne Vic. Australia; ^13^ Sydney School of Health Sciences The University of Sydney Camperdown NSW Australia; ^14^ HIST Health Research Institute and School of Allied Health University of Limerick Limerick Ireland; ^15^ Department of Speech, Language, Hearing: Sciences & Disorders University of Kansas Lawrence KS USA; ^16^ Department of Communication Sciences & Disorders University of Delaware Newark DE USA

## Abstract

Current methods for reporting interventions do not allow key questions of importance to practitioners, service providers, policy‐makers and people with DLD to be answered, and hence limit the implementation of effective interventions in the real world. To extend the existing *EQUATOR* guidelines to the context of speech language therapy/pathology for children with language disorder and to provide more specific guidance on *participants*, *interventions* and *outcomes* within the CONSORT checklist (used to improve the reporting of randomised controlled trials) and TIDieR (Template for Intervention Description and Replication) to ensure consistency of reporting. We will develop a core team to include representatives from each of the key groups who will either use or be influenced by the final reporting guidance across different countries. To achieve each set of aims, we will conduct reviews of the literature (which present typologies of intervention characteristics in (D)LD and related disorders); carry out focus groups; and use systematic consensus methods such as the Delphi technique, nominal group technique or consensus development conferences. Through the development and adoption of standard intervention reporting criteria, we anticipate that we will overcome the numerous barriers for practitioners, services and policy‐makers in applying intervention evidence to practice. We believe that establishing international consensus on reporting guidelines would significantly accelerate progress in DLD research and the ease with which it can be used in clinical practice, by capitalising on the growth in intervention studies to enable international collaboration and new methodologies of data pooling, meta‐analyses and cross‐study comparisons.

## Introduction

Developmental language disorder (DLD) is one of the most prevalent neurodevelopmental disorders (~8% at 5–6 years) with significant implications for the child or young person's daily life and risks of substantial negative consequences for health, education, employment, well‐being and social inclusion across the life course (Dubois, St‐Pierre, Desmarais, & Guay, 2020; Eadie et al., 2021; McKean et al., 2017). The provision of effective intervention, to mitigate such consequences, is a clear priority for educational and health services across the world. Recent decades have seen a burgeoning number of published intervention studies relevant to children with and at risk for DLD, revealing several approaches which have demonstrated efficacy. However, there are numerous barriers for practitioners, health and education services and policy‐makers in translating this evidence to practice, to service delivery design and to the allocation of funding. In this paper, we make the case that several of these barriers could be overcome through the development and adoption of standard intervention reporting criteria, and that now is the right time for their development. We end with an invitation to researchers, practitioners, parents/caregivers and people with DLD to join this endeavour.

### Barriers to be addressed to maximise the benefits of intervention research

The authors of this paper are an international group with expertise in speech and language pathology and in particular in child language identification, intervention practice and/or research. From these roles and our partnerships with practitioners, service providers, policy‐makers and people with DLD, we recognise that key questions of importance to these groups need to be answered. We know that many interventions work and can deliver change for children and young people with and at risk of DLD (Frizelle, Mullane, et al., 2021; Frizelle et al., 2021a; Greenwood, Schnitz, Carta, Wallisch, & Irvin, 2019; Law, Garrett, & Nye, 2004). Stakeholders now need answers as to which interventions work best, for which outcomes, for which children and with what dosage? For example, practitioners want to be able to make judgements as to whether study participants reflect their caseloads and to understand the ‘active ingredients’ of the interventions, so as to be able to tailor them to individual clients. Service providers want to know the most efficient dosage, context and skill mix to bring about change, and whether one approach is more effective than another. Policy‐makers want to be able to quantify the resource needed to create optimal outcomes, establish a minimally effective dose and identify the optimal ages for intervention. Lastly, children and young people with DLD and their families want to be able to choose interventions which address outcomes of value and importance to them and which can be delivered in ways which minimally disrupt their lives. To answer these questions, large scale, multi‐site and co‐ordinated intervention research programs as well as data synthesis and meta‐analytical methods are required. Despite the growing volume of DLD intervention research and improved reporting via the use of CONSORT guidelines, an opportunity is being missed to address critical questions from core stakeholders. It is not currently possible to extract conclusions relevant to the above questions from that evidence. It is our belief that these questions can only be answered through the development and adoption of standard intervention reporting criteria that would extend the existing *EQUATOR* guidelines to the context of SLT/P and provide more specific guidance on elements of CONSORT, thereby enabling meta‐analysis and comparison across studies.

### Barriers to be addressed to examine intervention dosage

In the context of financially constrained publicly funded services and the burden of intervention on families and children, intervention effectiveness needs to be viewed in parallel with efficiency, such that maximum outcomes are achieved with the minimal amount of time, access and affordability. One aspect of intervention central to the concept of efficiency is ‘dosage’, which includes both quantitative (how much? at what density? for how long?) as well as qualitative (in what form?) constructs. Two recent systematic reviews carried out by members of the Intervention Consensus for language disorder group (TICLD) examined and synthesised current evidence regarding optimal intervention dosage with respect to children with DLD (see Frizelle et al., 2021a, 2021b). The first review aimed to establish the degree to which the quantitative aspects of dosage (dose, dose frequency and total intervention duration) were specifically manipulated and compared in intervention studies and to draw conclusions regarding optimal dosage for phonology, vocabulary and morphosyntax outcomes. Using the definition coined by Warren, Fey, and Yoder (2007), dose is expressed as ‘the number of properly administrated teaching episodes during a single intervention session.’ (p. 71); dose frequency refers to the number of intervention sessions per unit of time; and total intervention duration is defined as the total period for which a given intervention is provided. However, when attempting to draw conclusions regarding optimal dosage, a number of issues arose in relation to the consistency and level of study detail reported. Variation in the literature regarding how *dose* is defined is problematic, with some authors applying the definition above, put forward by Warren et al. (2007), which focuses on teaching episodes and others defining dose as the total amount of time spent on a given target (e.g. Justice, Logan, Jiang, & Schmitt, 2017; Schmitt, Justice, & Logan, 2016). In addition, the subcomponents of dose posited by Warren are rarely specified. For example, while most studies specify the length of the intervention session, few report the average rate of teaching episodes per unit of time, the distribution of episodes within the session or the distribution of sessions over time. Some studies report on expressive dose (the number of times the child produces the target) but do not provide information on receptive dose (the number of times the child hears the target). Other studies specify planned dose but do not give detail on actual dose received and others extrapolate dose based on a percentage of the overall number of sessions. The concept of cumulative intervention intensity put forward by Warren et al. (2007) is also problematic and requires further refinement. It is currently defined as dose × dose frequency × total intervention duration. However, application of this formula does not distinguish between a frequency of once a week versus once a month (the integer would be 1 in both cases) and therefore cannot be uniformly applied. Differences in how intervention study procedures are reported and defined across studies make it very challenging to explore effectively how quantitative characteristics interact with other active ingredients (i.e., dose form) or to compare dose form mechanisms more definitively. Moreover, with such variability and lack of precision in reporting, meta‐analyses are not possible and only tentative conclusions can currently be drawn regarding the optimal dosage of interventions despite the large body of evidence available.

### Barriers to be addressed to examine dose form—the ‘active ingredients’ of interventions

The second review aimed to synthesise findings from studies in which dose form (the active ingredients of the intervention) was experimentally manipulated or statistically analysed (directly comparing one intervention to an alternative), while quantitative aspects of dosage were controlled. This allowed the authors to draw conclusions about optimal dose form and identify gaps in the evidence. Building on previous work by Warren et al. (2007) and Proctor‐Williams (2009), Frizelle et al. (2021a) put forward a taxonomy of active ingredients to be described in intervention studies, including *Techniques*—the specific actions/teaching behaviours thought to be of benefit or to effect change; *Procedures*—the combination and order of technique delivery; *Methods of Instruction*—whether the intervention is implemented implicitly, where with sufficient practice, children generate the particular rule or pattern themselves, or explicitly, where children are informed of the rule relevant to the teaching target; and *Intervention context*—to include three subcomponents—the activity within which the teaching episode is being delivered; whether the activity is primarily child‐centred or clinician‐directed and the degree of variability/similarity in the linguistic input or materials used.

As was the case with the first review, a number of reporting differences were highlighted, specifically in how dose form was described and in the levels of detail reported. Interventions that were similarly labelled used different techniques or procedures, and terms such as *prompting* or *cueing* were used inconsistently to describe a range of techniques, such as *imitation, questioning or sentence completion*, which were often not explicitly described (see Eisenberg, Bredin‐Oja, & Crumrine, 2020 for a review of variation within the single concept of imitation in how intervention can be delivered). It became evident that there is no agreed regulated practice for each dose form technique, and the level of detail in describing techniques is often insufficient to allow replication with fidelity. In addition, it was unclear whether certain techniques were implemented implicitly or with explicit methods of instruction. Reporting of contextual information was inconsistent and often missing despite the fact that context can interfere with, as well as facilitate children's learning (e.g. Kouri & Winn, 2006; Smeets et al., 2012). Contextual information regarding variability of the linguistic input and materials used was particularly scant, unless they were the manipulated variables. These limitations in reporting are problematic, as it is impossible for researchers or clinicians to know whether they are implementing a specific treatment approach with a degree of fidelity that will achieve results. Developing consensus on a standardised set of labels and definitions (with clear examples) will improve clarity regarding intervention components and ingredients; how those components are combined into procedures; and what the causal chain of action is in effecting change. The knowledge that intervention components will be consistently described by the same labels, each with a clear definition, will result in a shared language that can be used among professionals, their clients and families, and that will increase clarity with respect to SLT/P interventions that can be applied in a broader educational arena.

Given the complexity of language interventions for children and young people with DLD, which involve many interacting components, without precise and consistent descriptions of the nature and content of interventions, we cannot effectively translate them into practice.

### Barriers to be addressed to understand the population

Children with or at risk of D(LD) have always been a highly heterogeneous group. However, intervention studies tend to attract and recruit more advantaged demographic groups in a given population and may actively exclude participants with specific characteristics which have in the past been considered confounding, such as multilingualism, lower nonverbal IQ or associated diagnoses (Bishop, 2017) (REFS). Often participants in research studies do not represent those receiving or in need of treatment in the real world, making application to practice problematic. The adoption of DLD as a diagnostic term and framework brings with it a more inclusive approach to participant selection, in specifying that DLD can co‐occur with other neurodevelopmental disorders; a diagnosis of DLD does not require a mismatch between verbal and nonverbal ability; and that the presence of risk factors does not preclude a diagnosis of DLD. This more inclusive approach is to be welcomed. However, we believe that this, together with a focus on equity and social justice in SLT/P, brings with them the need to more thoroughly describe the nature of participant samples in intervention research along a number of key criteria. In this way, clinicians can understand if and how the study sample reflects their caseload or the specific client they are considering for this intervention; the research community can monitor the degree to which the diversity of the populations in need of SLT is truly represented and included in research; and we can develop the necessary data to enable meta‐analyses and subgroup analyses to determine whether intervention effects differ across key participant characteristics.

### Barriers to be addressed to understand outcomes

The choice of outcome measures across studies is almost as numerous as the studies themselves. How outcomes are defined, when they are measured and whether both target and generalisation items are included are all likely to have a substantive impact on study findings. Work specifying core outcomes has been recently undertaken in other communication disorder domains such as Aphasia (Wallace et al., 2021). However, we are unaware of any core outcomes set for DLD that has been developed in collaboration with relevant stakeholders. Outcomes of treatment studies almost exclusively relate to the specific language targets selected for treatment, rather than functional goals that may go beyond language to measure participation, quality of life etc. Some studies use bespoke measures, which may not be fully validated with regard to test–retest reliability and sensitivity to change. Other studies adopt change on diagnostic tools as evidence of treatment efficacy, despite the fact that these tools were developed for another purpose. Few studies report patient‐reported outcome measures [PROMS; e.g. the FOCUS (Washington et al., 2013)], which is unsurprising given the paucity of measures available for use with children. The development of an agreed minimum set of outcome domains which balance burden and comprehensiveness could allow comparisons across interventions, support interpretable meta‐analysis and ensure that we measure factors of importance to key stakeholders such as children with DLD themselves.

### Why now is the right time to develop consensus guidelines

The reporting inconsistencies highlighted in both reviews led the lead authors to think about the need for international consensus across several key intervention domains—active ingredients including techniques, procedures etc.; quantitative intervention dosage; study participants; and outcome measures. We believe that establishing international consensus on language intervention reporting guidelines would significantly accelerate progress in DLD research and translation, capitalising on the growth in intervention studies to enable international collaboration and new methodologies of data pooling, meta‐analyses and cross‐study comparisons. The growth in implementation science tools, such as the behaviour change technique taxonomy (Michie, Van Straten, & West, 2011), consensus methodologies in healthcare research and outcome measurement and emerging SLT/P intervention taxonomies (e.g. Denman, Kim, Munro, Speyer, & Cordier, 2021) provide clear models and methods to support our aims. They also bring a sense of urgency. The development of numerous, competing taxonomies would only serve to muddy the waters of an already rather murky pool of research evidence and delay transfer into practice. *International consensus* is necessary if we are to realise the potential benefits described above.

### A call for action and an invitation to shape the agenda

In autumn of 2021, Cristina McKean and Pauline Frizelle convened a discussion group of international experts who had either carried out SLT/P intervention research or systematic reviews in this area. These core group members represent Australia, Finland, Ireland, the United Kingdom and the United States and are co‐authors of this publication. The group have met three times over the course of 6 months and have discussed and prioritised aspects of intervention to consider for reporting guideline development, specifically:

- Intervention characteristics—mapping roughly on to the concept of dosage put forward by Warren et al. (2007) and modified by Frizelle et al. (2021a) (including qualitative and quantitative aspects of dose), so that the hypothesised ‘active ingredients’ of an intervention are adequately described.
- Participant characteristics—to include demographic and diagnostic information.
- Core outcome set—developed through methods informed by COMET guidelines.

Our overarching aim is to extend the existing *EQUATOR* guidelines (Enhancing the QUAlity and Transparency Of health Research) to the context of speech language therapy/pathology for children with D(LD) and to provide more specific guidance on elements of CONSORT (used to improve the reporting of randomised controlled trials) (Schulz et al., 2010) and TIDieR (Template for Intervention Description and Replication) (Hoffmann et al., 2014) to ensure consistency of reporting. In particular, we aim to address aspects of *participants*, *interventions* and *outcomes* within the CONSORT checklist and the *what*, *how*, *where* and *when and how much* within the TIDieR checklist. In addition, we aim to achieve international acceptance of these guidelines. We plan to

- develop a set of *intervention characteristics* reporting guidelines which are
  - applicable to an extensive range of SLT/P interventions for children with and at risk of (D)LD
  - agreed by an international consensus to describe and name active ingredients and quantitative aspects of dosage
  - clearly named and defined and recognisable across countries
  - distinct, comprehensive, precise and non‐overlapping
  - categorised for ease of use
- develop a set of *participant characteristics* reporting guidelines which
  - accurately describe participants
  - enable meta‐analysis and data pooling to examine effects of differing participant characteristics on intervention efficacy
  - enable clinicians, managers and policymakers accessing research publications to consider the applicability of findings to the clinical caseloads/populations they serve and their translation into practice
  - improve readers/reviewers/editor's ability to judge bias in recruitment and sampling strategies with respect to issues of social justice, and to audit publication practices with respect to representation—driving up equity in research and supporting judgements of external validity.
- develop a core set of outcome measure reporting guidelines which
  - are agreed by an international consensus
  - are clearly defined across countries, indicating the linguistic, communicative and cognitive domains to measure
  - are hierarchical and distinguish between target and generalisation items
  - provide recommendations with respect to outcome measurement timing and its relevance to children's learning
  - are relevant and important to all key stakeholders—to include functional impact.
- Create an overarching document which (checklist with explanation/definition document) includes each of these guideline subsets.

If these standard reporting guidelines are to deliver the benefits we hope for, of enabling cross‐study comparisons, meta‐analysis, data pooling, international application and addressing issues of social justice, it is essential that key stakeholders shape and engage with this programme of work. Our priority is that the resulting set of guidelines address the priorities of people with (D)LD and their families, are meaningful to clinicians; practicable to researchers; internationally and cross‐culturally relevant; and ethical in relation to burden on individuals with or at risk of (D)LD and their families. We do not underestimate this challenge. In keeping with previous work carried out by Denman et al. (2021) to develop a broad taxonomy to describe language interventions, we anticipate that the development of intervention characteristic reporting guidelines will be the most challenging. In particular, establishing agreement on the names and definitions of specific intervention techniques. To do this, we need to develop a core team to include representatives from each of the key groups who will use and be influenced by the final reporting guidance across different countries, that is, individuals with (D)LD, their parents/carers, clinicians working with individuals with or at risk of (D)LD; researchers developing and investigating intervention effects; journal editorial boards and policymakers. To achieve each set of aims, we will conduct focus groups with all of the aforementioned stakeholder groups, as well as using systematic consensus methods such as the Delphi technique, nominal group technique or consensus development conferences.

This paper serves as a call to action among key stakeholders, for whom this work matters. We invite members of each of the key groups to contact us to signal their interest in being involved and to specify in what capacity. Our goals will only be achieved if the work we do is truly international and so the authorship team aim to work with national professional bodies and through social media to reach our key stakeholders across a range of countries. To that end, we invite those who wish to be involved and to support this work to contact us through the form on our webpage (ticld@ucc.ie) and look out for events on social media over the coming months.

Join us! If we can work together and harness the collective benefits of intervention research worldwide, we believe we can achieve so much more to improve the lives of children with or at risk of (D)LD.

**Key points**

- Current methods for reporting interventions are inconsistent in both the level of study detail and in how different intervention components are described and therefore limit the implementation of these interventions in the real world.
- Extending the existing *EQUATOR* guidelines to the context of speech language therapy/pathology for children with (D)LD and providing more specific guidance on *participants*, *interventions* and *outcomes* within the CONSORT and TIDieR checklists will ensure a new level of consistency in the reporting of interventions for children with and at risk of D(LD).
- Through the development and adoption of standard intervention reporting criteria, we will overcome many of the current barriers for practitioners, in translating intervention evidence to practice.
